# Supplementary material for: Shear stress switches the association of endothelial enhancers from ETV/ETS to KLF transcription factor binding sites
Source: Sci Rep. 2022 Mar 21;12:4795. doi: 10.1038/s41598-022-08645-8 (PMC8938417; doi:10.1038/s41598-022-08645-8)
Supplement: Supplementary file 2 — Supplementary Figures. [file 41598_2022_8645_MOESM2_ESM.pdf]

A

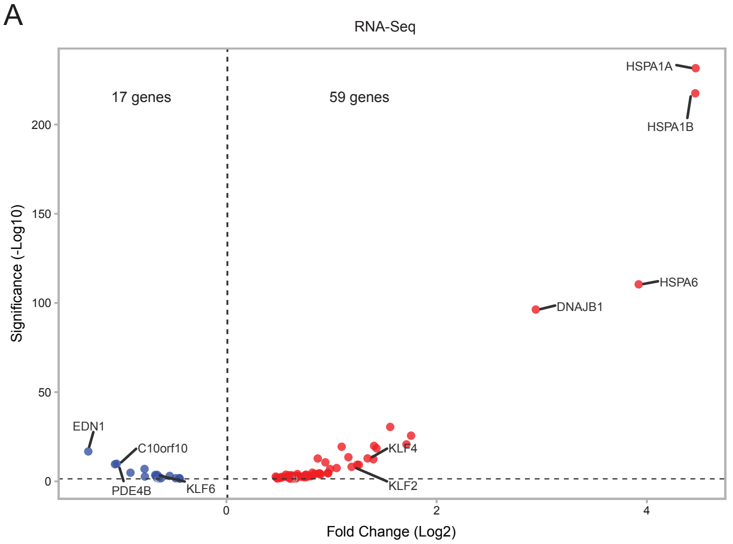

B

ENSEMBL Gene ID	log2FoldChar	lfcSE	padj	Gene Name
ENSG00000204389	4.46393816	0.13601455	2.61×10^{-232}	HSPA1A
ENSG00000204388	4.46031914	0.14027444	3.00×10^{-218}	HSPA1B
ENSG00000173110	3.92204737	0.17237454	3.77×10^{-111}	HSPA6
ENSG00000132002	2.94345393	0.13841216	4.96×10^{-97}	DNAJB1
ENSG00000013441	1.75667656	0.1557838	2.42×10^{-26}	CLK1
ENSG00000117616	1.71267223	0.16759484	1.96×10^{-21}	RSRP1
ENSG00000120694	1.55819638	0.12728521	3.14×10^{-31}	HSPH1
ENSG00000124216	1.42812358	0.1475603	3.15×10^{-19}	SNAI1
ENSG00000151929	1.40452248	0.1402296	1.37×10^{-20}	BAG3
ENSG00000188483	1.39806471	0.17346433	4.31×10^{-13}	IER5L
ENSG00000136826	1.34308432	0.1634457	1.35×10^{-13}	KLF4
ENSG00000160570	1.26020551	0.17783703	5.81×10^{-10}	DEDD2
ENSG00000170345	1.24456699	0.17488521	4.92×10^{-10}	FOS
ENSG00000127528	1.19094644	0.17743925	7.73×10^{-09}	KLF2
ENSG00000004478	1.1605387	0.13809586	3.04×10^{-14}	FKBP4
ENSG00000080824	1.09653281	0.11079645	4.03×10^{-20}	HSP90AA1
ENSG00000204569	1.04857835	0.16180978	3.51×10^{-08}	PPP1R10
ENSG00000197019	0.98590745	0.15698868	1.19×10^{-07}	SERTAD1
ENSG00000140403	0.96888764	0.18088438	2.56×10^{-05}	DNAJA4
ENSG00000198576	0.96312538	0.1795659	2.55×10^{-05}	ARC

C

ENSEMBL Gene ID	log2FoldChar	lfcSE	padj	Gene Name
ENSG00000078401	-1.3139386	0.14209871	1.78×10^{-17}	EDN1
ENSG00000184588	-1.0601802	0.14694164	2.53×10^{-10}	PDE4B
ENSG00000165507	-1.0438904	0.14302887	1.45×10^{-10}	C10orf10
ENSG00000137331	-0.9125793	0.16691337	1.48×10^{-05}	IER3
ENSG00000139318	-0.7791677	0.12388776	1.17×10^{-07}	DUSP6
ENSG00000116717	-0.7754477	0.17784408	0.002150288	GADD45A
ENSG00000067082	-0.6769533	0.13959285	0.000274993	KLF6
ENSG00000156030	-0.6722485	0.14918951	0.001220766	ELMSAN1
ENSG00000124575	-0.6585195	0.1700084	0.014378774	HIST1H1D
ENSG00000118515	-0.6583668	0.13477376	0.000236013	SGK1
ENSG00000177352	-0.6435607	0.18037964	0.041628795	CCDC71
ENSG00000113369	-0.6403809	0.15122304	0.003450353	ARRDC3
ENSG00000143067	-0.6215881	0.16299926	0.017798993	ZNF697
ENSG00000163788	-0.5397281	0.11818853	0.000950745	SNRK
ENSG00000185112	-0.4830494	0.12738184	0.019104008	FAM43A
ENSG00000143878	-0.448177	0.11611076	0.014962019	RHOB
ENSG00000124766	-0.4432221	0.12282025	0.036081265	SOX4

Tsaryk et al., Supplementary Fig. 1

A

ENSEMBL Gene ID	log2FoldChar	lfcSE	padj	Gene Name
ENSG00000164530	7.23157246	0.5280092	1.17×10^{-39}	PI16
ENSG00000104783	6.32277206	0.57912565	5.64×10^{-25}	KCNN4
ENSG00000136999	5.85304792	0.41122402	9.33×10^{-43}	NOV
ENSG00000154734	5.75946696	0.36967531	4.36×10^{-51}	ADAMTS1
ENSG00000159871	5.70060654	0.52796033	1.93×10^{-24}	LYPD5
ENSG00000174600	5.59695605	0.3902341	2.59×10^{-43}	CMKLR1
ENSG00000165272	5.55498339	0.4965064	3.05×10^{-26}	AQP3
ENSG00000138061	5.35585517	0.37750583	1.59×10^{-42}	CYP1B1
ENSG00000095752	5.20564682	0.55119064	1.27×10^{-18}	IL11
ENSG00000127528	5.18998818	0.32922041	3.58×10^{-52}	KLF2
ENSG00000136826	5.18397413	0.31076796	2.35×10^{-58}	KLF4
ENSG00000138623	5.10866282	0.36068163	2.01×10^{-42}	SEMA7A
ENSG00000115461	4.81144856	0.57753478	1.69×10^{-14}	IGFBP5
ENSG00000138670	4.66333256	0.45288322	3.40×10^{-22}	RASGEF1B
ENSG00000156427	4.59630516	0.61176361	9.00×10^{-12}	FGF18
ENSG00000163273	4.54553639	0.6422798	1.97×10^{-10}	NPPC
ENSG00000159167	4.51412326	0.64154326	2.56×10^{-10}	STC1
ENSG00000103196	4.44455529	0.50811566	5.73×10^{-16}	CRISPLD2
ENSG00000158955	4.35011621	0.57866494	8.81×10^{-12}	WNT9B
ENSG00000136153	4.20294978	0.30668654	1.14×10^{-39}	LMO7

B

ENSEMBL Gene ID	log2FoldChar	lfcSE	padj	Gene Name
ENSG00000120738	-4.4450289	0.50319511	2.71×10^{-16}	EGR1
ENSG00000131386	-4.295856	0.44044927	6.88×10^{-20}	GALNT15
ENSG00000157404	-4.1179487	0.36338554	6.98×10^{-27}	KIT
ENSG00000165507	-4.0506711	0.34476833	6.69×10^{-29}	C10orf10
ENSG00000091879	-3.8945261	0.33828627	9.34×10^{-28}	ANGPT2
ENSG00000185950	-3.5480676	0.58826512	1.22×10^{-07}	IRS2
ENSG00000121966	-3.4732088	0.64951835	4.69×10^{-06}	CXCR4
ENSG00000088756	-3.4616065	0.32997433	4.63×10^{-23}	ARHGAP28
ENSG00000185477	-3.3086477	0.63290089	8.26×10^{-06}	GPRIN3
ENSG00000196421	-3.1331681	0.52521289	1.72×10^{-07}	LINC00176
ENSG00000175746	-3.0777502	0.39842478	1.91×10^{-12}	C15orf54
ENSG00000118407	-3.0379694	0.64954491	0.000106491	FILIP1
ENSG00000171408	-3.0088392	0.36997637	8.22×10^{-14}	PDE7B
ENSG00000198355	-2.9427072	0.31875833	7.75×10^{-18}	PIM3
ENSG00000005187	-2.9318501	0.36192126	1.05×10^{-13}	ACSM3
ENSG00000128594	-2.8804755	0.55544442	1.02×10^{-05}	LRRC4
ENSG00000137266	-2.8721365	0.38840704	2.11×10^{-11}	SLC22A23
ENSG00000151967	-2.8712331	0.44830357	1.38×10^{-08}	SCHIP1
ENSG00000004799	-2.8600017	0.45302171	2.42×10^{-08}	PDK4
ENSG00000152207	-2.8474613	0.70002554	0.001222303	CYSLTR2

C

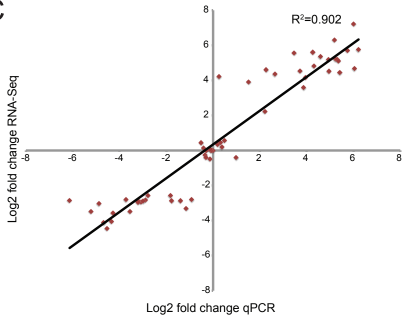

E

Genes	Our data	Maleszewska et al		Maimari et al		Dekker et al	
	DE genes	DE genes	Overlap	DE genes	Overlap	DE genes	Overlap
Upregulated	647	615	233	1003	249	600	111
Downregulated	367	835	104	635	71	439	44
Total	1014	1450	350	1638	330	1039	163
%			34.5%		32.5%		15.7%

D

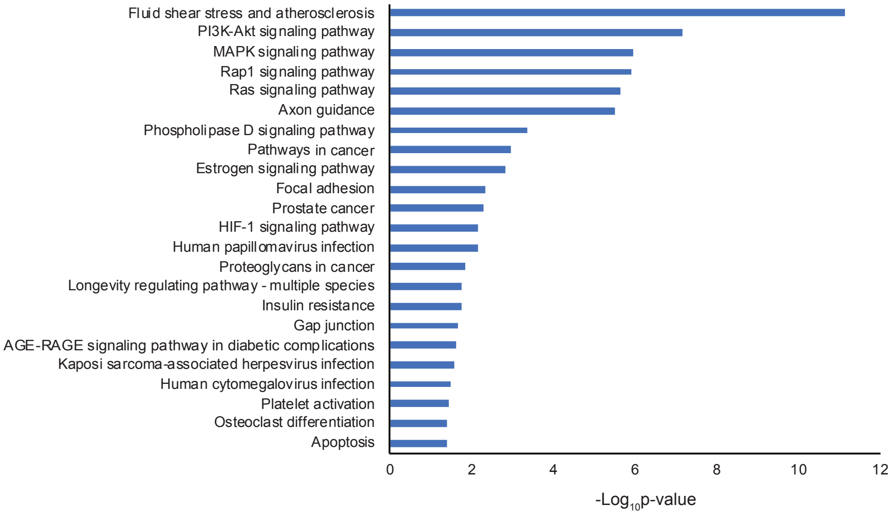

Tsaryk et al., Supplementary Fig. 2

A

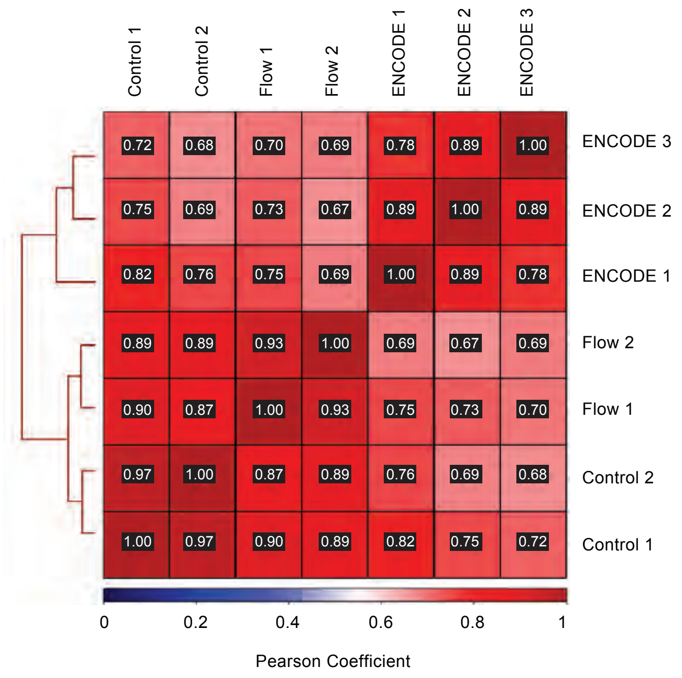

B

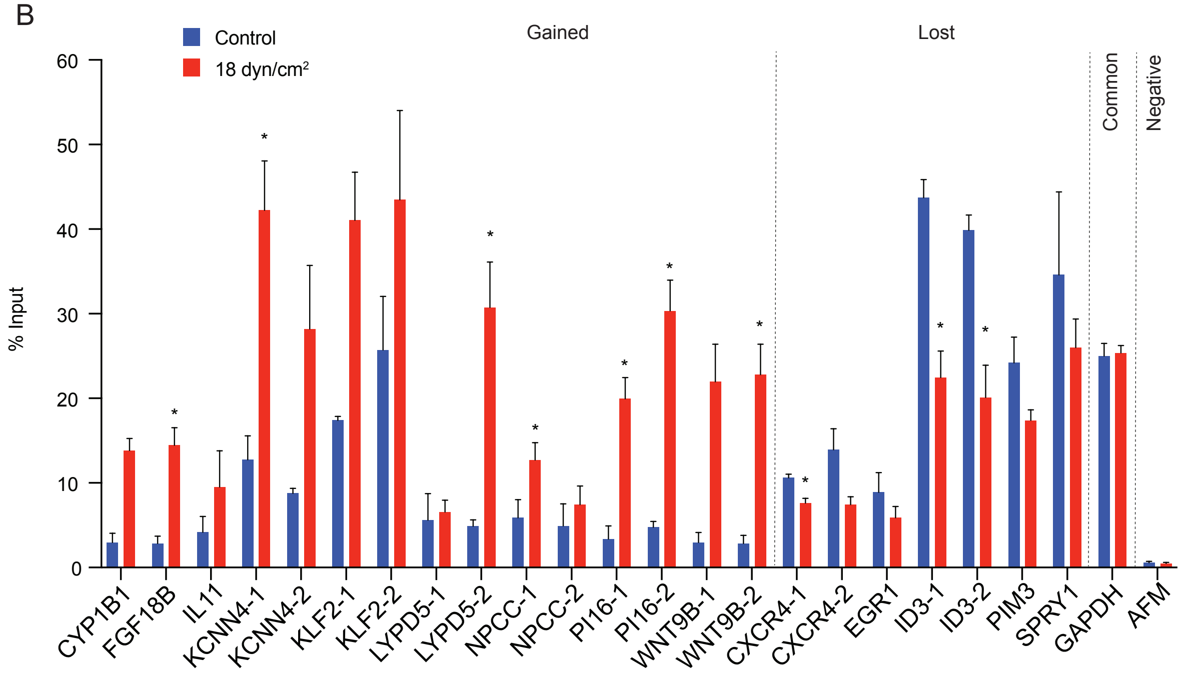

Tsaryk et al., Supplementary Fig. 3

A

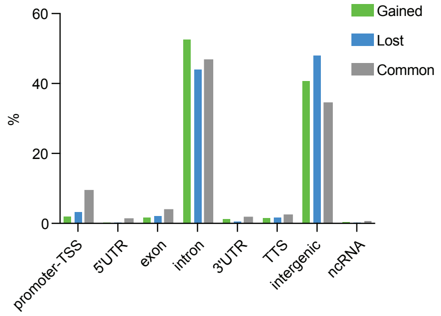

B

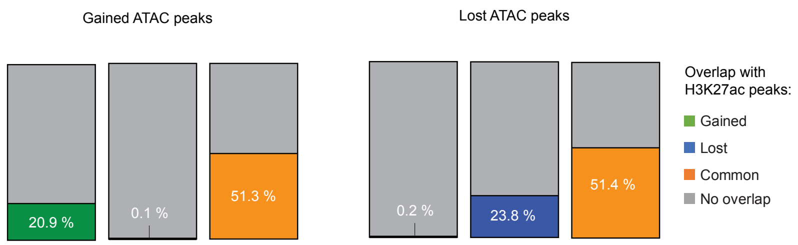

C

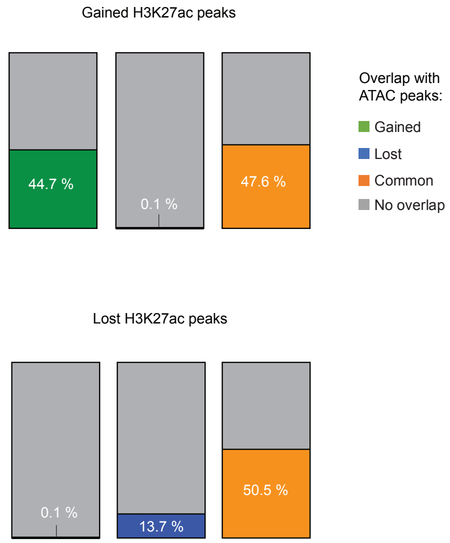

D Novel motifs in Gained ATAC/Gained H3K27ac peaks

Motif	Best Match	p-value
TGGGTGTGGC	Klf1/MA0493.1/Jaspar(0.933)	10^{-122}
CCCTTCCTGC	ETS1(ETS)/Jurkat-ETS1-ChIP-Seq(GSE17954)/ Homer(0.815)	10^{-24}
GATTTGACACAC	BATF::JUN/MA0462.1/Jaspar(0.606)	10^{-20}
GAAGTCTGGATG	PB0060.1_Smad3_1/Jaspar(0.625)	10^{-18}
TGATTCAC	Fra1(bZIP)/BT549-Fra1-ChIP-Seq(GSE46166)/ Homer(0.860)	10^{-18}
CAGTATCAATCA	PB0144.1_Lef1_2/Jaspar(0.798)	10^{-18}
TCATGACAGA	MAFG::NFE2L1/MA0089.1/Jaspar(0.723)	10^{-16}
GGAAGGGGAGGA	SP2/MA0516.1/Jaspar(0.679)	10^{-15}
AGACATCAGC	c-Jun-CRE(bZIP)/K562-cJun-ChIP-Seq(GSE31477)/ Homer(0.699)	10^{-15}
AGAGGTTATT	COUP-TFII(NR)/Artia-Nr2f2-ChIP-Seq(GSE46497)/ Homer(0.692)	10^{-15}

E Novel motifs in Lost ATAC/Lost H3K27ac peaks

Motif	Best Match	p-value
TTCCTGTTTA	Ets1-distal(ETS)/CD4+-PolII-ChIP-Seq(Barski_et_al.)/ Homer(0.852)	10^{-31}
AAAACAAT	Sox5/MA0087.1/Jaspar(0.887)	10^{-16}
ATAAACAGCCTT	Foxf1(Forkhead)/Lung-Foxf1-ChIP-Seq(GSE77951)/ Homer(0.637)	10^{-16}
ACTTTTTCTTTG	PB0093.1_Zfp105_1/Jaspar(0.744)	10^{-15}
AAACATCAGG	ZBTB18/MA0698.1/Jaspar(0.758)	10^{-15}
TGATCCCCAAGA	MZF1/MA0056.1/Jaspar(0.671)	10^{-12}
AAGATTATGTTT	PB0016.1_Foxj1_1/Jaspar(0.671)	10^{-12}

F Known motifs in Common ATAC/Gained H3K27ac peaks

Motif	Motif Name	p-value
CTGGGTGTGGCC	EKLF(Zf)/Erythrocyte-Klf1-ChIP-Seq(GSE20478)	10^{-5}
TTGACCTTTG	RARa(NR)/K562-RARa-ChIP-Seq(Encode)	10^{-5}
GATGAGTCATCC	Fosl2(bZIP)/3T3L1-Fosl2-ChIP-Seq(GSE56872)	10^{-4}
AAGGTCACCGTGACC	ERE(NR),IR3/MCF7-ERa-ChIP-Seq	10^{-4}
GATGACTCAT	JunB(bZIP)/DendriticCells-Junb-ChIP-Seq(GSE36099)	10^{-4}
GCCACACCCA	Klf4(Zf)/mES-Klf4-ChIP-Seq(GSE11431)	10^{-4}
CCACTTCCTGTT	Etv2(ETS)/ES-ER71-ChIP-Seq(GSE59402)	10^{-4}
GGTCAAAGGTCA	COUP-TFII(NR)/K562-NR2F1-ChIP-Seq(Encode)	10^{-4}
AGGGTGTGGC	KLF5(Zf)/LoVo-KLF5-ChIP-Seq(GSE49402)	10^{-4}
GGGGGGGG	Maz(Zf)/HepG2-Maz-ChIP-Seq(GSE31477)	10^{-4}

G Known motifs in Common ATAC/Lost H3K27ac peaks

Motif	Motif Name	p-value
ATTTCCTGTTTT	ETV1(ETS)/GIST48-ETV1-ChIP-Seq(GSE22441)	10^{-21}
AAACAACG	Ets1-distal(ETS)/CD4+-PolII-ChIP-Seq(Barski_et_al.)	10^{-19}
CCCTAACTTACT	ERG(ETS)/VCaP-ERG-ChIP-Seq(GSE14097)	10^{-18}
CAGTGGAAAT	GABPA(ETS)/Jurkat-GABPa-ChIP-Seq(GSE17954)	10^{-16}
CAAGGGCACT	SPDEF(ETS)/VCaP-SPDEF-ChIP-Seq(SRA014231)	10^{-16}
CTATAAACTCTG	ETS1(ETS)/Jurkat-ETS1-ChIP-Seq(GSE17954)	10^{-15}
TGGCATGTTGCA	Etv2(ETS)/ES-ER71-ChIP-Seq(GSE59402)	10^{-14}
TTTCTCCACGCA	ETV4(ETS)/HepG2-ETV4-ChIP-Seq(ENCODE)	10^{-11}
CCTGAAATTGTT	EWS:ERG-fusion(ETS)/CADO_ES1-EWS:ERG-ChIP-Seq (SRA014231)/	10^{-11}
AGTCTGGGATTC	Fli1(ETS)/CD8-FLI-ChIP-Seq(GSE20898)	10^{-10}

Tsaryk et al., Supplementary Fig. 4

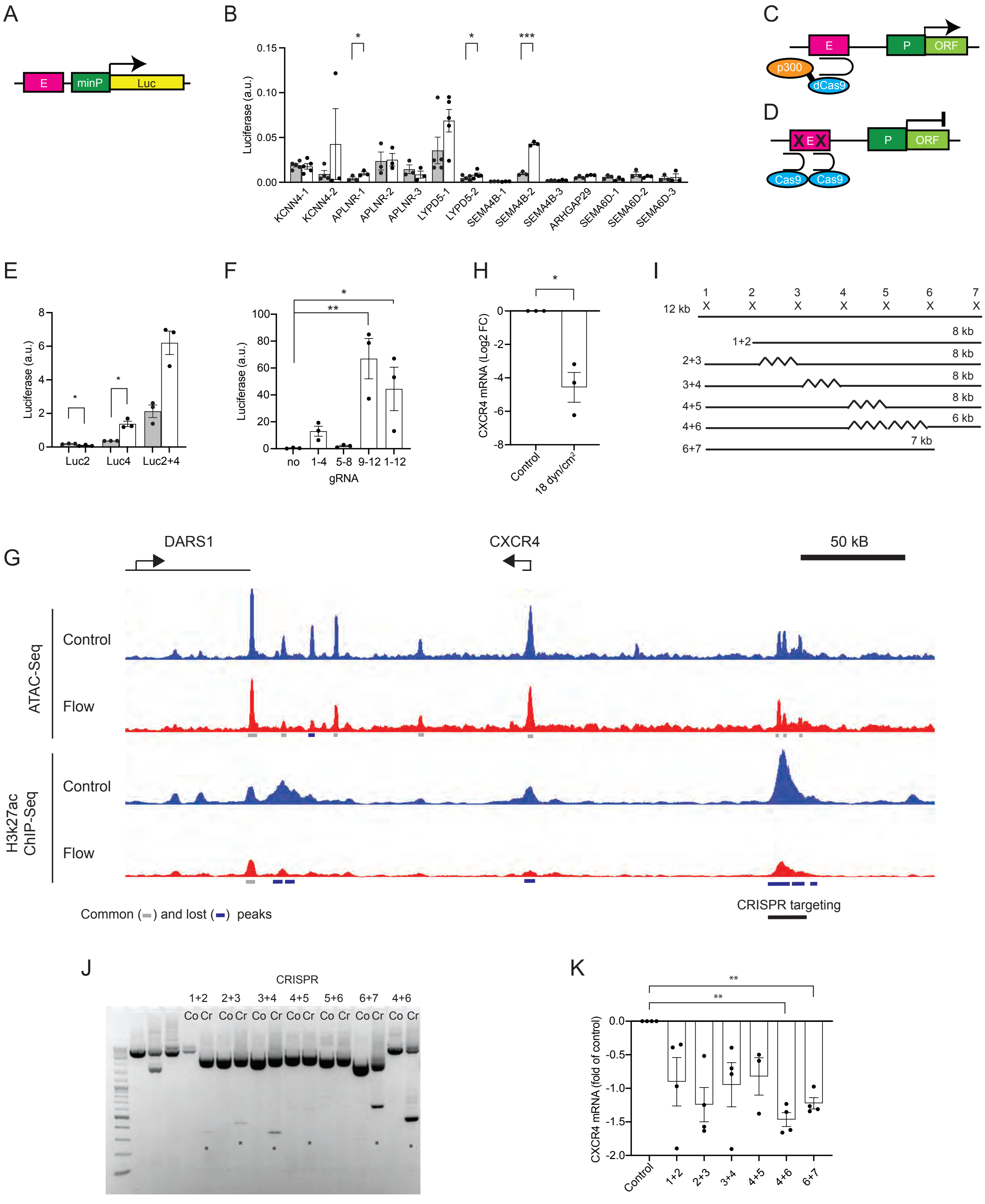

Tsaryk et al., Supplementary Fig. 5

A

B

1.61×10^{-13}

A

ENSEMBL Gene ID	log2FoldChar	lfcSE	padj	gene_name
ENSDARG00000052646	3.42153839	0.35031736	1.03×10^{-19}	ftr69
ENSDARG00000039943	3.06727405	0.24633644	1.27×10^{-31}	fam46ba
ENSDARG00000098155	2.95610381	0.28914694	1.44×10^{-21}	si:dkeyp-3b12.11
ENSDARG00000019874	2.56354094	0.31665277	1.61×10^{-13}	hsph1
ENSDARG00000037099	2.53560502	0.26666363	1.19×10^{-18}	irs2a
ENSDARG00000038489	2.49300998	0.22164013	5.47×10^{-26}	b3gnt7
ENSDARG00000075833	2.44780036	0.23852428	1.07×10^{-21}	lyve1a
ENSDARG00000041022	2.39342709	0.24258267	4.48×10^{-20}	pdcd4b
ENSDARG00000060113	2.38844383	0.21944098	2.11×10^{-24}	znf395a
ENSDARG00000098925	2.29118385	0.30319	9.07×10^{-12}	prdm1b
ENSDARG00000096518	2.23143627	0.35066543	2.20×10^{-8}	si:ch73-95a24.1
ENSDARG00000069282	2.18169268	0.3028213	1.12×10^{-10}	bbc3
ENSDARG00000099002	2.14552934	0.31031123	7.16×10^{-10}	creb5a
ENSDARG00000101135	2.13959387	0.31773682	2.18×10^{-9}	si:dkey-85k7.7
ENSDARG00000027529	2.13089445	0.24266781	6.23×10^{-16}	hmox1a
ENSDARG00000024746	2.1286108	0.39057968	3.26×10^{-6}	hsp90aa1.2
ENSDARG00000036848	2.0836956	0.17989377	1.55×10^{-27}	slc43a2a
ENSDARG00000023217	2.02945464	0.19637621	5.68×10^{-22}	crema
ENSDARG00000020761	2.01762514	0.22426926	1.03×10^{-16}	arrdc2
ENSDARG00000094557	2.01254617	0.32863327	9.16×10^{-08}	nupr1

B

ENSEMBL Gene ID	log2FoldChar	lfcSE	padj	gene_name
ENSDARG00000053204	-1.9815825	0.28403354	4.82×10^{-10}	snx22
ENSDARG00000061923	-1.9630544	0.16346249	1.47×10^{-29}	amotl2a
ENSDARG00000040764	-1.829106	0.19509112	3.97×10^{-18}	id1
ENSDARG00000078800	-1.6770781	0.3338926	2.50×10^{-5}	slc26a10
ENSDARG00000037555	-1.6720874	0.3033587	2.41×10^{-6}	atoh8
ENSDARG00000042934	-1.659224	0.23592535	3.34×10^{-10}	ctgfa
ENSDARG00000018441	-1.5575572	0.1724045	7.62×10^{-17}	heg1
ENSDARG00000024204	-1.523809	0.22797693	2.98×10^{-9}	mcm3
ENSDARG00000045748	-1.4951781	0.18453174	1.60×10^{-13}	stab2
ENSDARG00000061948	-1.4915171	0.24854555	1.85×10^{-7}	amotl2b
ENSDARG00000031894	-1.4695724	0.23481427	4.08×10^{-8}	lef1
ENSDARG00000099395	-1.4649494	0.33276355	3.35×10^{-4}	cables1
ENSDARG00000101707	-1.4281017	0.34863545	1.06×10^{-3}	si:ch211-156b7.4
ENSDARG00000030104	-1.4200317	0.25455302	1.76×10^{-6}	sh3bp4
ENSDARG00000035694	-1.4188867	0.32617363	4.05×10^{-4}	stm
ENSDARG00000038785	-1.417025	0.19775843	1.46×10^{-10}	abcf2a
ENSDARG00000002216	-1.3707023	0.2790729	4.09×10^{-5}	tbx3a
ENSDARG00000090337	-1.3600781	0.22591338	1.66×10^{-7}	pprc1
ENSDARG00000012506	-1.3427077	0.29865342	2.36×10^{-4}	rnpep
ENSDARG00000038703	-1.3397517	0.31008385	4.53×10^{-4}	hkdc1

C

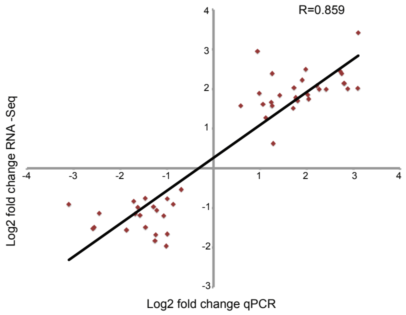

D

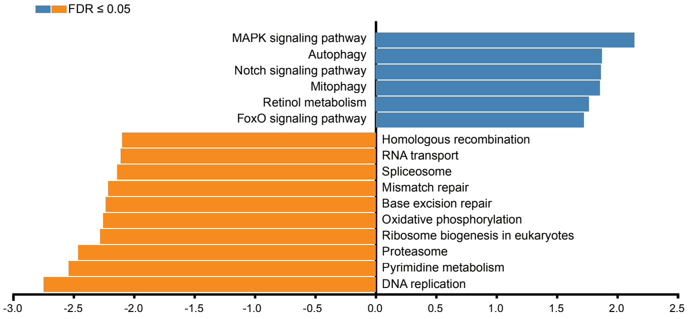

E

H. sapiens		D. rerio		Overlap	
DE genes	Nr. of genes	DE genes	Nr. of genes	Nr. of genes	%
UP	647	DOWN	483	30	6.2
DOWN	367	UP	584	47	8.0
ALL	1014	ALL	1067	146	13.7

Tsaryk et al., Supplementary Fig. 6

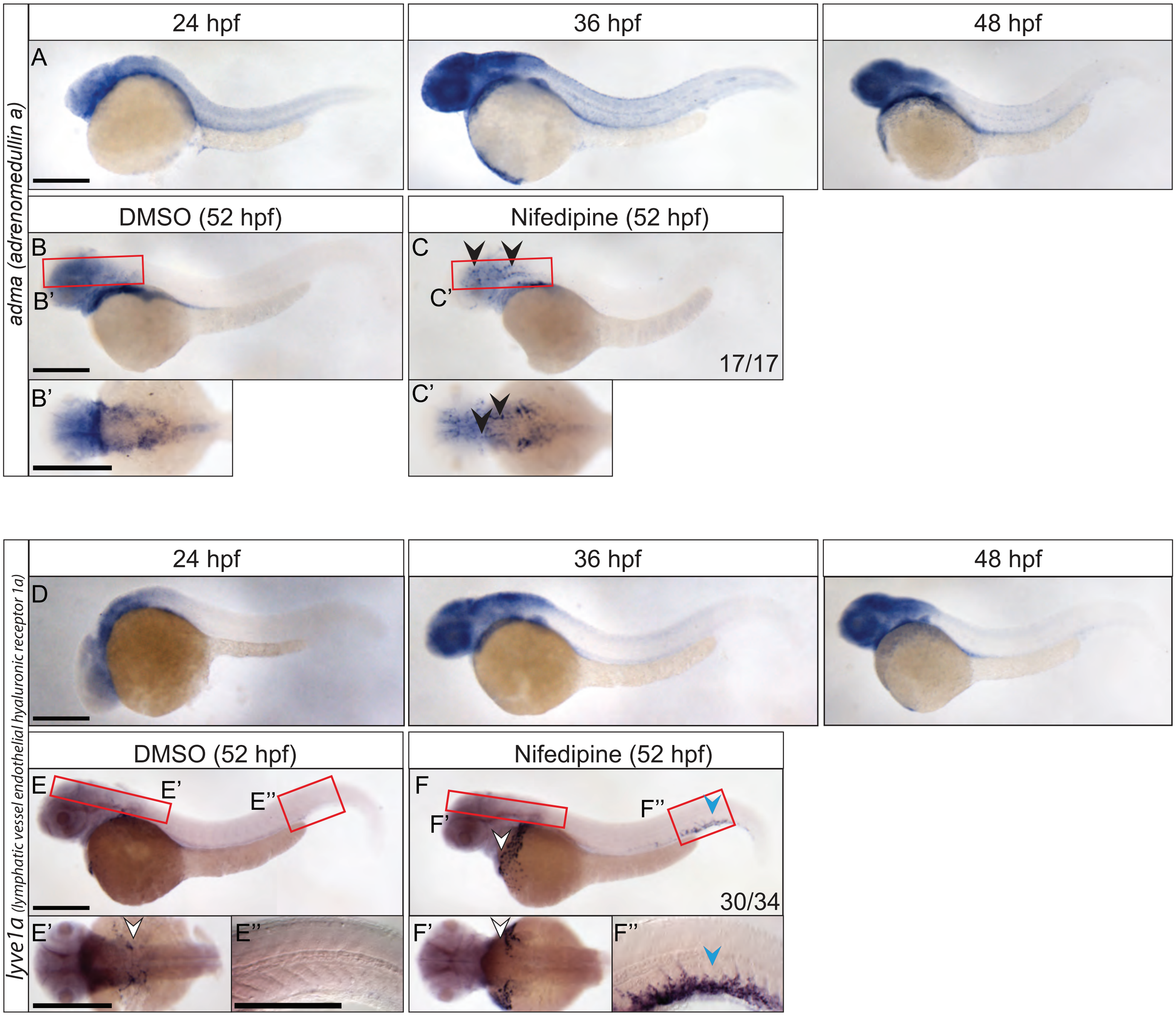

Tsaryk et al., Supplementary Fig. 7

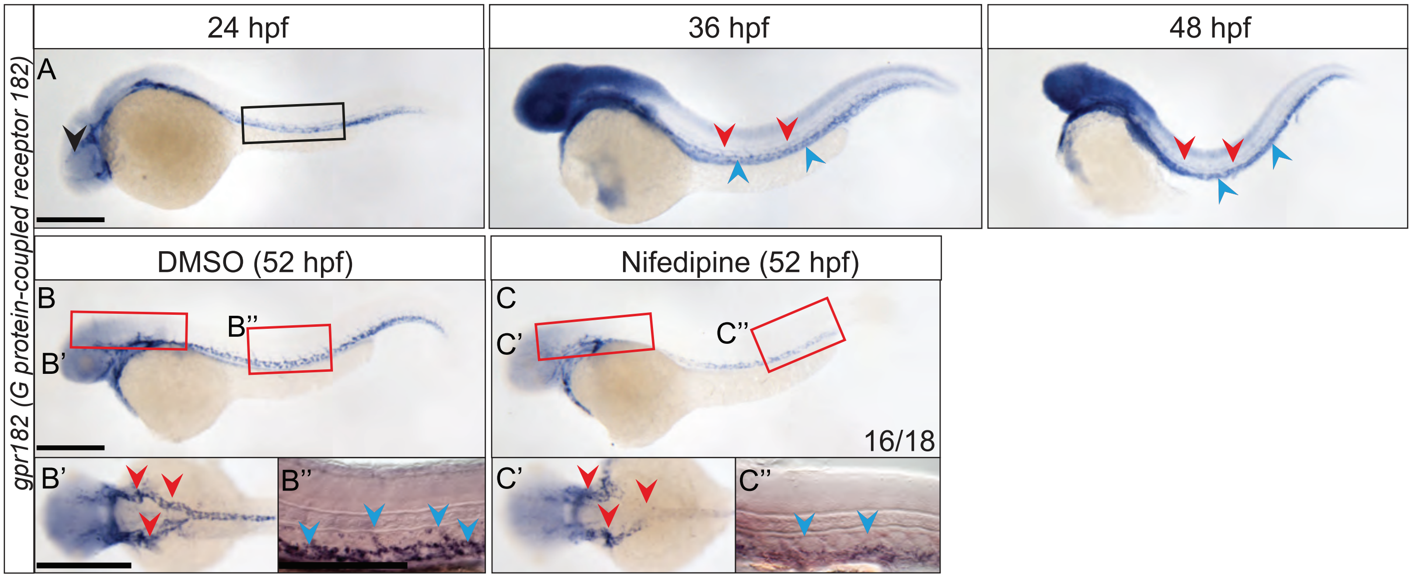

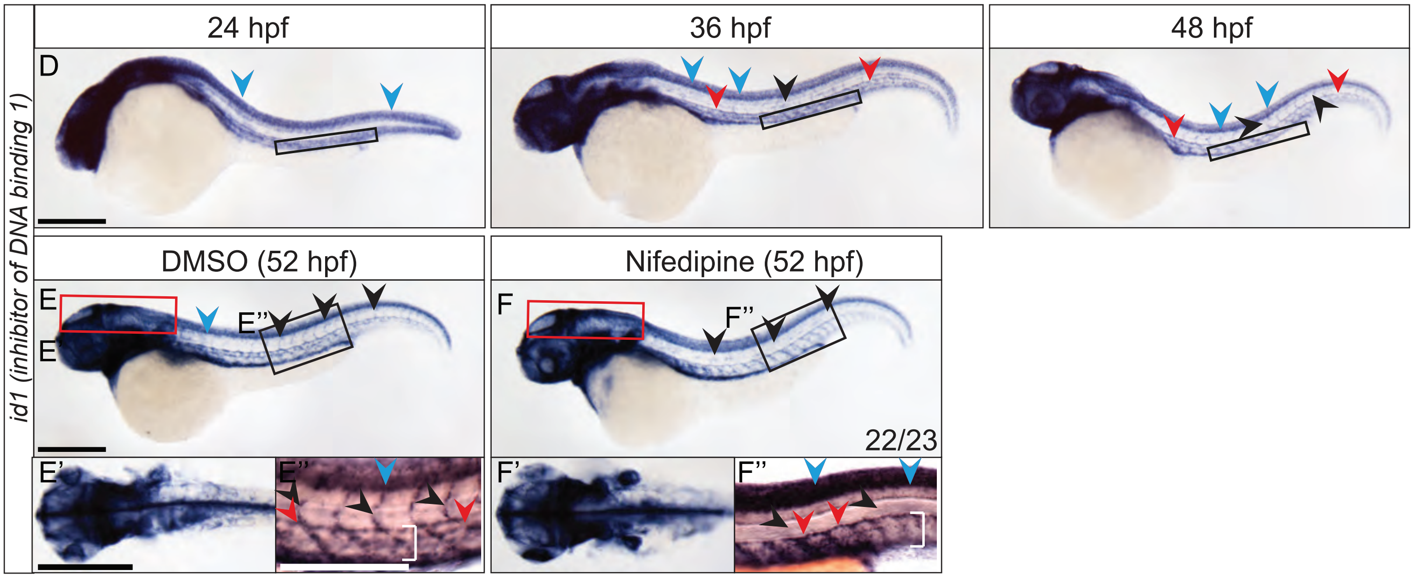

Tsaryk et al., Supplementary Fig. 8

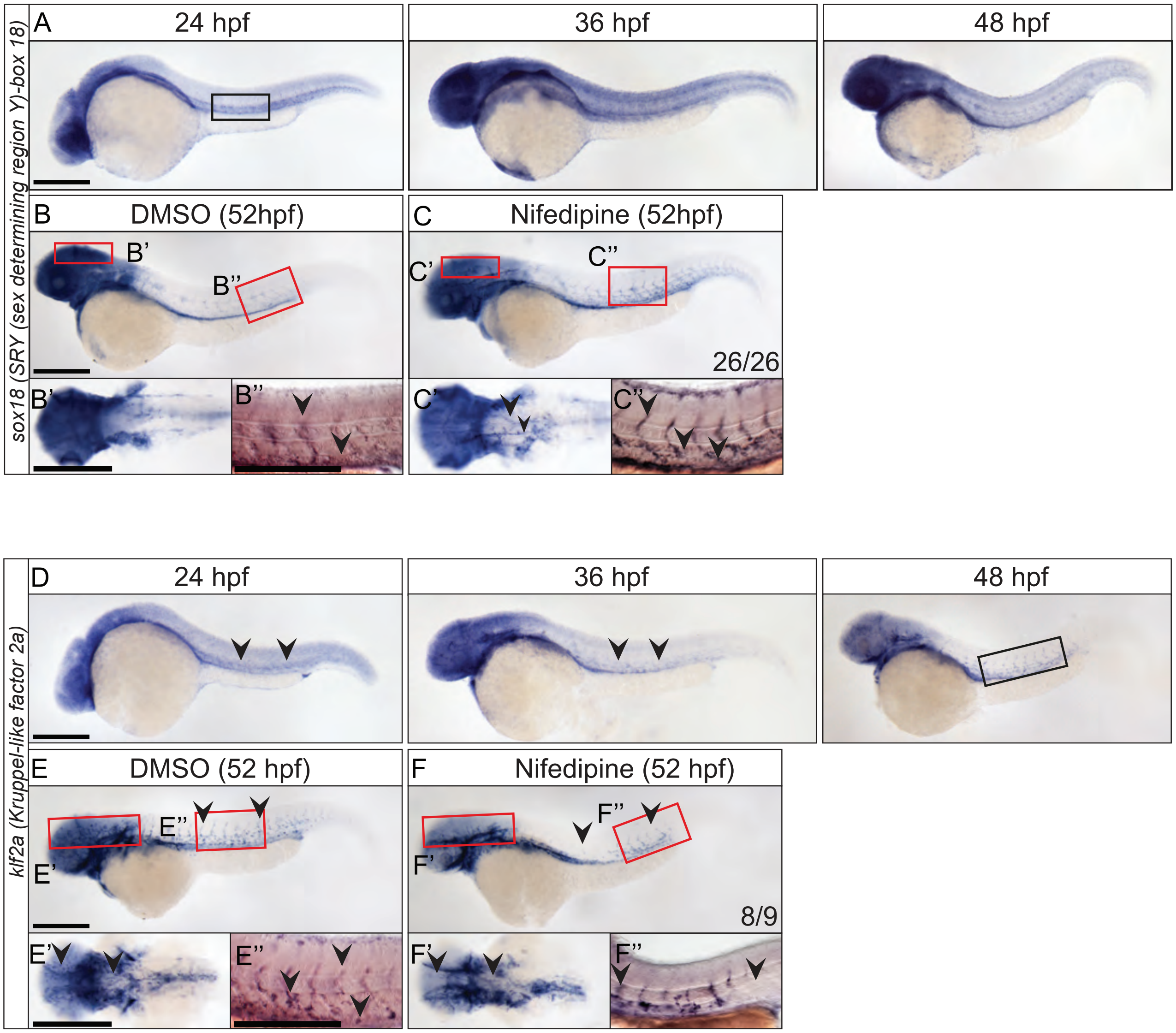

Tsaryk et al., Supplementary Fig. 9

uncropped agarose gel picture related to Fig. 3G

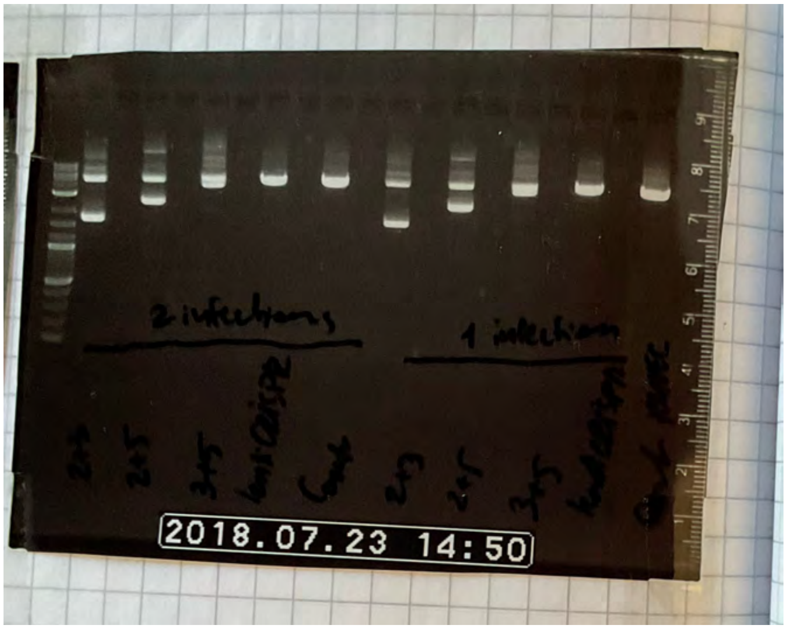

uncropped agarose gel picture related to Supplementary Fig. 5J

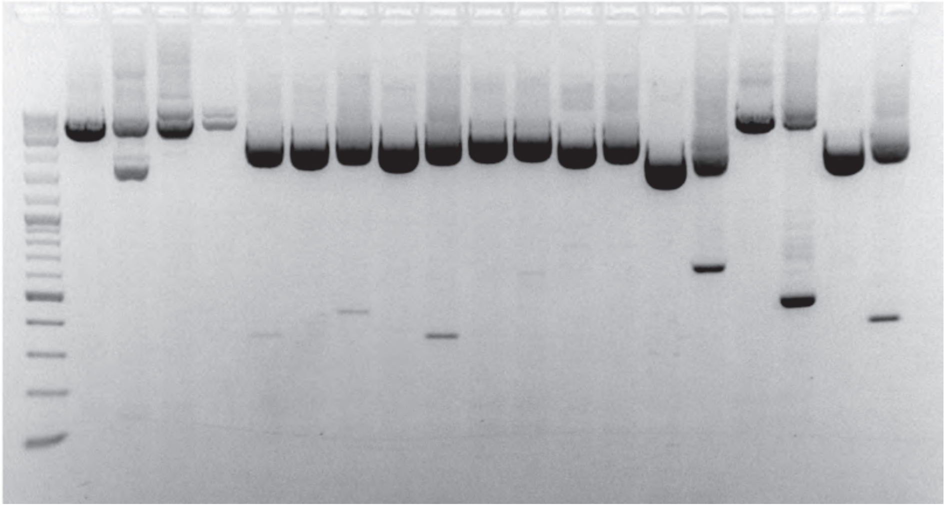

Supplementary Figure 1. Exposing HUVEC to shear stress for 30 minutes regulates the expression of a distinct set of genes. A. Volcano plot representing differentially expressed (DE) genes (FDR < 0.05) upon HUVEC exposure to shear stress of 18 dyn/cm^2 for 30 min. Upregulated genes are labelled in red and downregulated genes in blue. **B, C**. Top 20 upregulated genes (**B**) and 17 genes downregulated in HUVEC exposed to shear stress for 30 minutes (**C**).

Supplementary Figure 2. Overlap of shear stress regulated genes between different studies. A, B. 20 genes most up- (**A**) or downregulated (**B**) upon 6 h exposure to 18 dyn/cm^2 shear stress (RNA-Seq results). **C**. Correlation of mRNA expression measured by RNA-Seq and qPCR in shear stress exposed HUVEC (n =2). **D**. Over-representation (ORA) analysis of KEGG pathways regulated in the RNA-Seq dataset. **E**. Comparison of DE genes regulated by shear stress in this study with published datasets. RNA-Seq data of Maleszewska et al. (HUVEC exposed to 20 dyn/cm^2 for 72 h), microarray data of Dekker et al. (HUVEC with adenoviral overexpression of KLF2 for 7 days) and metaanalysis of multiple microarray studies (Maimari et al.) are listed. The amount of up- and down-regulated genes overlapping with our data is presented. The total amount of overlapping genes (irrespective of up- or down-regulation) is additionally shown as percentage compared to the total number of DE genes in our RNA-Seq.

Supplementary Figure 3. **Verification of H3K27ac ChIP-Seq data. A.** Pairwise correlation analysis of ChIP-Seq HUVEC samples (cultured under static, “Control”, or shear stress conditions, “Flow”) with HUVEC H3K27ac ChIP-Seq from ENCODE. Pearson coefficient values for each comparison are represented. **B**. Verification of selected gained, lost and common H3K27ac ChIP-Seq peaks with ChIP-qPCR. Additionally, a region with no H3K27ac ChIP-Seq enrichment was tested as negative control. The data are presented as % of input (mean + sem, n = 3, * p < 0.05 compared to corresponding control, two-tailed paired t-test).

Supplementary Figure 4. **Comparison of ATAC-Seq and H3K27ac ChIP-Seq data sets and DNA motif analysis. A.** Annotation of all gained, lost and common ATAC-Seq peaks to the defined regions of the genome. **B**. Overlap of gained and lost ATAC-Seq peaks with gained, lost and common H3K27ac ChIP-Seq peaks. Numbers represent percentage of overlap. **C.** Overlap of

gained and lost H3K27ac ChIP-Seq peaks with gained, lost and common ATAC-Seq peaks. Numbers represent percentage of overlap. **D, E**. de novo motif analysis of DNA motifs enriched in gained (**D**) or lost (**E**) ATAC-Seq peaks that overlap with gained and lost H3K27ac ChIP-Seq peaks, respectively. Most enriched motifs (top 10 for gained and all 7 for lost) and corresponding best match are shown. **F, G**. Known motifs enriched in common ATAC-Seq peaks that overlap with gained (**F**) or lost (**G**) H3K27ac-ChIP-Seq peaks. The 10 most enriched motifs are shown.

Supplementary Figure 5. Functional analysis of flow responsive enhancers. A. Schematic representation of luciferase constructs with a putative enhancer (E) upstream of a minimal promoter (minP) and firefly luciferase gene (Luc). **B**. Luciferase assays with constructs carrying putative enhancers associated with DE genes in HUVEC exposed to 18 dyn/cm^2 for 24 h (data is presented as arbitrary units (a.u.) after normalization to the signal of renilla luciferase; mean ± sem, n = 3 - 5, * $p < 0.05$, **** $p < 0.001$, compared to a corresponding static control, two-tailed paired t-test). **C**. Schematic representation of dCas9-p300 construct designed to target putative enhancers to affect gene expression. **D**. Schematic representation of the removal of a putative enhancer (E) with Cas9 and two specific gRNAs (P, promoter; ORF, open reading frame). **E**. Luciferase assay showing luciferase activity of Luc2 and Luc4 constructs, as well as a construct containing both regions (Luc2+4) in HUVEC exposed to 18 dyn/cm^2 for 24 h (the data is presented as arbitrary units (a.u.) after normalization to the signal of renilla luciferase; mean ± sem, n = 3, * $p < 0.05$, two-tailed paired t-test). **F.** Luciferase activity of Luc4 construct targeted by dCas9-p300 and specific gRNAs in HEK293T17 cells (the data are presented as arbitrary units (a.u.) after normalization to the signal of a renilla luciferase construct; mean ± sem, n = 3, * $p < 0.05$, ** $p < 0.01$, one-way ANOVA with Dunnet's multiple comparison test). **G**. Normalized ATAC-Seq and H3K27ac ChIP-Seq tracks around the CXCR4 gene. The bars below ATAC-Seq and H3K27ac ChIP-Seq tracks represent corresponding common (grey) or lost (blue) peaks. **H**. CXCR4 mRNA expression in HUVEC exposed to 18 dyn/cm^2 for 6 h (Log2 fold of control, mean ± sem, n = 3, * $p < 0.05$, two-tailed paired t-test). **I**. Schematic representation of the genomic region upstream of the CXCR4 gene and the pairs of CRISPR/Cas9 constructs designed to remove parts of the region. **J**. Ethidium bromide gel confirming removal of targeted genomic regions. PCR products indicated with an asterisk are shorter than in corresponding controls (Co) and result from successful CRISPR targeting (Cr). Uncropped agarose gel is shown

in Suppl. Fig. 10. **K**. CXCR4 mRNA expression in HUVEC infected with lentiviral constructs encoding Cas9 and gRNAs targeting genomic regions upstream of the CXCR4 gene (data are shown as fold of control, mean ± sem, n = 4, ** $p < 0.01$, repeated measures one-way ANOVA with Dunnet's multiple comparison test).

Supplementary Figure 6. Analysis of flow regulated genes in zebrafish. A. 20 genes most up- (**A**) or downregulated (**B**) in zebrafish ECs 4 h after blocking blood flow (48 – 52 hpf, RNA-Seq results). **C**. Correlation of mRNA expression measured by RNA-Seq and qPCR in zebrafish endothelial cells after blocking blood flow (n =2). **D**. Gene set enrichment analysis (GSEA) of KEGG pathways regulated in the zebrafish RNA-Seq dataset. **E**. Overlap of up- and downregulated, as well as all DE genes in zebrafish with human orthologues regulated by shear stress.

Supplementary Figure 7. Analysis of genes downregulated by flow. A. Whole mount in situ hybridization for *adma* shows no detectable vascular expression in 24, 36 and 48 hpf zebrafish embryos. **B, C**. Nifedipine treatment from 48 to 52 hpf induces *adma* expression in brain blood vessels (black arrowheads) **C**, enlarged in **C'**), while expression is undetectable in DMSO controls (**B**, enlarged in **B'**). **D**. Whole mount hybridization for *lyve1a* showing no vascular expression in 24, 36 and 48 hpf embryos. **E, F**. *lyve1a* expression in 52 hpf embryos after 4 h of treatment with DMSO (**E**) or nifedipine (**F**). Enlarged images of the head (**E'** and **F'**) and the trunk (**E''** and **F''**) regions highlighted by red rectangles in **E** and **F**. Flow block induces an increased *lyve1a* expression in the common cardinal vein (white arrowheads in **E**', **F** and **F'**) and in caudal plexus (blue arrowheads in **F** and **F''**). Numbers represent number of embryos with depicted expression pattern out of the total number of embryos tested. Scale bar is 300 μm.

Supplementary Figure 8. Analysis of genes upregulated by flow. A. Whole mount in situ hybridization for *grp182* in 24, 36 and 48 hpf zebrafish embryos. *grp182* is expressed in trunk axial vasculature (black box) and in the primordial midbrain channel (black arrowhead) at 24 hpf and in the dorsal aorta (red arrowheads) and posterior cardinal vein (blue arrowheads) at 36 and 48 hpf. **B, C**. *grp182* expression in 52 hpf embryos after 4 h treatment with DMSO (**B**) or nifedipine (**C**). Enlarged images of the head (**B'** and **C'**) and the trunk (**B''** and **C''**) regions

highlighted by red rectangles in **B** and **C**. Flow block decreases *grp182* expression in lateral dorsal aorta (red arrowheads) and posterior cardinal vein (blue arrowheads). **D**. Whole mount in situ hybridization for *id1* in 24, 36 and 48 hpf zebrafish embryos. Expression of *id1* is detected in the neural tube (blue arrowheads), hypochord (red arrowheads), axial vasculature (black box) and intersegmental blood vessels (black arrowheads). **E, F**. *id1* expression in 52 hpf embryos after 4 h treatment with DMSO (**E**) or nifedipine (**F**). Enlarged images of the head (**E'** and **F'**) and the trunk (**E''** and **F''**) regions highlighted by red and black rectangles in **E** and **F**. Flow block reduces *id1* expression in intersegmental vessels (black arrowheads) and in axial vessels (white brackets), while expression in the neural tube (blue arrowheads) and hypochord (red arrowheads) remains unaffected. Numbers represent number of embryos with depicted expression pattern out of the total number of embryos tested. Scale bar is 300 μm.

Supplementary Figure 9. Regulation of *sox18* and *klf2a* expression by flow in zebrafish embryos. **A**. Whole mount in situ hybridization for *sox18* in 24, 36 and 48 hpf zebrafish embryos. The black box highlights vascular expression in the trunk at 24 hpf, which is reduced at later stages. **B, C**. *sox18* expression in 52 hpf embryos after 4 h treatment with DMSO (**B**) or nifedipine (**C**). Enlarged images of the head (**B'** and **C'**) and the trunk (**B''** and **C''**) regions highlighted by red rectangles in **B** and **C**. Black arrowheads indicate vascular expression, which is increased after inhibition of blood flow. **D**. Whole mount in situ hybridization for *klf2a* in 24, 36 and 48 hpf zebrafish embryos. Black arrowheads indicate expression in trunk axial vessels at 24 and 36 hpf and the black box highlights increased expression in axial vessels, as well as in intersegmental vessels at 48 hpf. **E, F**. *klf2a* expression in 52 hpf embryos after 4 h treatment with DMSO (**E**) or nifedipine (**F**). Enlarged images of the head (**E**' and **F**') and the trunk (**E**'' and **F**'') regions highlighted by red boxes in **E** and **F**. Black arrowheads indicate vascular expression, which is decreased after flow block in the brain and in the trunk. Numbers represent number of embryos with depicted expression pattern out of the total number of embryos analyzed. Scale bar is 300 μm.

Supplementary Figure 10. Uncropped agarose gels for Fig. 3G and Suppl. Fig. 5J.
